# Serotonergic Control of Spine Morphology During Growth in Zebrafish

**DOI:** 10.64898/2026.09.20.753056

**Published:** 2026-09-22

**Authors:** Stevana R. Schauer, Bryson Tyler B. Ricamona, Maximilian R. Segeberg, Laura Desban, Zane L. Walsh, Judith S. Eisen, Daniel T. Grimes

**Affiliations:** 1 Institute of Molecular Biology, Department of Biology University of Oregon Eugene, OR United States; 2 Institute of Neuroscience, Department of Biology University of Oregon Eugene, OR United States

## Abstract

Scoliosis is a three-dimensional deformity of the vertebral column that commonly emerges during growth, yet the biological mechanisms that initiate and drive curve progression remain poorly understood. Serotonin (5-hydroxytryptamine [5-HT]) has long been linked to scoliosis through its relationship with pineal melatonin signaling and through genetic associations with human disease, but its role in spinal morphogenesis has remained unclear. Here, using zebrafish, we show that serotonergic signaling is required for normal spine morphology. Transient exposure to 5-HT during development induces later spinal abnormalities, while loss of Tph2, the principal enzyme required for neuronal 5-HT synthesis, causes progressive three-dimensional spinal curvature during juvenile growth, mimicking aspects of adolescent idiopathic scoliosis (AIS). *tph2* mutants undergo initially normal vertebral patterning while targeted ablation of the pineal gland does not induce scoliosis, arguing against a pineal or early skeletal origin for the 5-HT-associated spinal curves. The Reissner fiber, which has been linked to spinal curvature in zebrafish, also formed normally in *tph2* mutants. Instead, mutants showed a marked reduction in Fev-expressing intraspinal serotonergic neurons together with increased locomotor activity. Mutants exhibited longer swim bouts and shorter periods of inactivity between bouts. Importantly, these motor defects were evident shortly before the onset of overt spinal curvature.. Together, these findings identify neuronal serotonin as a critical regulator of spinal stability and support a model in which altered serotonergic control of motor function changes the mechanical environment experienced by the growing spine, increasing its susceptibility to progressive curvature.

## RESULTS AND DISCUSSION

5-HT is a particularly intriguing candidate in scoliosis because it lies at the intersection of two potential mechanisms for spinal curvature. First, 5-HT is closely linked to pineal gland function, where it serves both as a biosynthetic precursor of melatonin and as a local signal that promotes melatonin synthesis (Lee et al., 2021). Melatonin signaling and the pineal gland have long been linked to scoliosis through human genetic studies (Qiu et al., 2006) and through pinealectomy experiments in several vertebrate models, in which removal of the pineal gland resulted in spinal curvature (Pflugfelder, 1953; Machida et al., 1993; O’Kelly et al., 1999; Man et al., 2014). Second, 5-HT is a major modulator of spinal motor circuits that control locomotion and posture (Brustein et al., 2003; Gabriel et al., 2009; Montgomery et al., 2018). Altered neural control of posture has been proposed to contribute to AIS (Herman et al., 1985; Catanzariti et al., 2014; Dufvenberg et al., 2018), while individuals with AIS also exhibit differences in gait and movement, including altered pelvic motion and paraspinal muscle activation (Kim et al., 2020; Paramento et al., 2024). Because locomotor activity continually imposes mechanical forces on the growing vertebral column, altered serotonergic control of these circuits therefore provides a plausible route through which 5-HT could influence spinal alignment. Consistent with a possible role for 5-HT in scoliosis, variants in *TPH1*, which encodes an enzyme required for 5-HT synthesis, have been associated with AIS susceptibility and with continued curve progression despite brace treatment (Wang et al., 2008; Xu et al., 2011). However, these associations have not been consistently replicated across populations, leaving their significance uncertain (Takahashi et al., 2011; Li et al., 2021). More broadly, it is challenging to distinguish whether altered postural, locomotor, or muscular differences in patients contribute to curve initiation or arise secondarily after scoliosis has developed. We therefore used zebrafish, an established model of scoliosis (Hayes et al., 2014; Grimes et al., 2016; Boswell and Ciruna, 2017), to test directly whether serotonergic signaling is required for normal spine morphology.

### The 5-HT synthesis enzyme, Tph2, is required for normal zebrafish spine morphology

To determine whether serotonergic signaling can influence spine morphology, we exposed wild-type zebrafish larvae to exogenous 5-HT by adding 1 or 2 mM 5-HT to the rearing water from the 1-cell stage until 6 days post fertilization (pf). Animals were then raised to 2.5 months pf for skeletal analysis by X-ray imaging. Mock treated zebrafish exhibited normal spine morphology and well-formed vertebrae. In contrast, fish treated with 5-HT showed spinal curves and apparent vertebral defects at both concentrations tested (Fig. 1A; 5/16 for 1 mM 5-HT; 4/15 for 2 mM 5-HT), in addition to craniofacial abnormalities, consistent with the established role of serotonergic signaling in craniofacial development (Reisoli et al., 2010). Thus, transient exposure to 5-HT during development can disrupt later vertebral and spinal morphology.

We next assessed the consequences of reduced serotonergic signaling. Tryptophan hydroxylase 2 (Tph2) is the principal enzyme required for neuronal 5-HT synthesis and is expressed throughout the zebrafish central nervous system, including the spinal cord (Bellipanni et al., 2002; Montgomery et al., 2016). We therefore asked whether genetic perturbation of 5-HT synthesis through loss of Tph2 is sufficient to disrupt spinal morphology.

We raised homozygous *tph2* mutants to adulthood and found they exhibited conspicuous body dysmorphology, including prominent kinks near the dorsal and caudal fins (Fig. 1B; Oikonomou et al., 2019). Three-dimensional reconstructions of micro-computed tomography (μCT; Bearce et al., 2023) scans revealed significant spinal curves in *tph2* mutants (Fig. 1C-C’). To quantify spinal shape, we compared the dorsoventral and mediolateral positions of successive vertebrae along the spine (Voigt et al., 2026). In the dorsoventral plane, wild-type fish showed the expected smooth dorsal curve. By contrast, *tph2* mutants showed markedly greater dorsoventral displacement across the vertebral column (Fig. 1D; *P* = 4.72 × 10^-4^, permutation test). This effect was driven by vertebrae 12–22, which were significantly displaced after correction for multiple comparisons.

Next, we assessed curvature in the mediolateral direction (Fig. 1D’). Because lateral curves occurred in either direction, with no left or right bias, we compared absolute mediolateral vertebral displacement. Mutants exhibited substantially greater lateral displacement than wild-type fish (*P* = 3.15 × 10^-5^, permutation test). Unlike the dorsoventral phenotype, the mediolateral difference extended across broader regions of the vertebral column and was strongest through the middle and posterior spine.

Because scoliosis in humans and several zebrafish models shows sex-dependent differences, we compared curvature between male and female *tph2* mutants. We detected no sex-dependent differences in either dorsoventral (P = 0.254) or mediolateral curvature (P = 0.889; permutation tests), indicating that the phenotype affects both sexes similarly. Together, these findings show that Tph2 is required for normal spinal alignment in zebrafish.

### Spinal curvature appears during juvenile growth and precedes vertebral malformations in *tph2* mutants

When raised to 1 year of age, all *tph2* mutants examined developed spinal curvature (> 100). To determine when curvature first emerges, we monitored *tph2* mutants and wild-type siblings through the first 91 days pf. Mutants grew at the same rate as wild-type animals (*P* = 0.936, two-way ANOVA) and spinal curves first became detectable at approximately 20 days pf, then increased in penetrance during juvenile growth, reaching 71% by 91 days pf (Fig. 2A-B). Mediolateral curvature generally preceded dorsoventral curvature, indicating that the three-dimensional phenotype develops progressively rather than appearing simultaneously in both anatomical planes.

We next asked whether spinal curvature in *tph2* mutants originates from abnormal vertebral development. In adult mutants, three-dimensional μCT reconstructions revealed localized vertebral abnormalities within regions of curvature (Fig. 2C-D and **Videos 1–2**). Individual vertebrae were variably compressed, warped, rotated, and appeared fused in some severely affected adults (Fig. 2C-D). Quantitative analysis of isolated vertebrae revealed alterations in vertebral morphology within curved regions of *tph2* mutant spines (Fig. 2E). Vertebral shape was assessed by measuring rostral-caudal length and dorsoventral height on opposing sides of each vertebral body and calculating length (L1/L2) and height (H1/H2) aspect ratios (Bearce et al., 2022). Values close to 1 therefore indicate a symmetric vertebra, whereas increasing deviation from 1 reflects greater vertebral distortion. Compared with wild-type vertebrae, *tph2* mutants showed a significantly greater mean displacement of the length aspect ratio from 1 (Welch’s *t*-test, *P* = 0.0142), whereas displacement of the height aspect ratio was not significantly different (*P* = 0.292), possibly due to increased variability in this metric in controls. When length and height measurements were combined into a single metric, distortion was significantly increased in *tph2* mutants (*P* = 0.0339). Thus, spinal curvature in *tph2* mutants is accompanied by modest vertebral distortion within curved regions of the spine.

These malformations could initiate spinal curvature or arise secondarily as the spine deforms. To distinguish between these possibilities, we examined vertebral development before and during curve onset. We crossed *tph2* mutants to TgBAC(*entpd5a:KillerRed)*, which labels mineralizing domains of the notochord sheath that subsequently give rise to vertebral centra (Wopat et al., 2018). From 7 to 21 days pf, during the period when vertebral patterning and mineralization are occurring, *tph2* mutants showed normal segmentation and regularly spaced mineralizing domains indistinguishable from those of wild-type siblings (Fig. 2F). We therefore found no evidence that abnormal early vertebral patterning precedes the onset of spinal curvature.

We next examined vertebral morphology immediately after curves first became apparent using high resolution μCT. Vertebrae from heterozygous siblings were morphologically normal, whereas *tph2* mutants exhibited localized vertebral warping at the curve apex (**Video 3**). Importantly, these animals did not yet show the vertebral fusions observed in severely affected adults. Small mineralized fragments were occasionally visible between distorted vertebrae (Fig. 2G), consistent with early structural remodeling or damage accompanying the developing curve.

Together, these findings indicate that *tph2* mutants undergo initially normal vertebral patterning before developing spinal curvature during juvenile growth. Local vertebral distortion appears around the time that curves emerge, while more severe malformations, including occasional vertebral fusions, develop later within chronically curved regions. The timing of curve onset and subsequent vertebral remodeling parallels AIS, in which spinal curvature emerges during growth and vertebral wedging and rotation can develop progressively with the deformity.

### The pineal gland and Reissner fiber do not mediate *tph2*-dependent spinal curvature

We next asked which serotonergic populations are responsible for maintaining spine morphology. The pineal gland, a major site of melatonin synthesis in which 5-HT serves as a biosynthetic precursor, has long been implicated in scoliosis. Beginning with Pflugfelder’s 1953 observation that surgical pinealectomy induced spinal curvature in guppies, pineal removal has subsequently been associated with scoliosis-like deformities in several vertebrate models (Pflugfelder, 1953; Thillard et al., 1959; Machida et al., 1993; Kanemura et al., 1997; O’Kelly et al., 1999; Cheung et al., 2005; Man et al., 2014). These findings led to the longstanding hypothesis that pineal-derived factors, particularly melatonin and 5-HT, contribute to spinal stability (Bagnall et al., 1996; Cheung et al., 2003; Girardo et al., 2011; Machida et al., 1995, 1996; Moreau et al., 2004; Turgut et al., 2003; Wang et al., 1998). However, the effects of pinealectomy have varied between studies and species, leaving the potential role of the pineal in spine morphology unresolved. Nevertheless, *tph2* is expressed in the zebrafish pineal gland (Teraoka et al., 2004), raising the possibility that its role in spine morphology could be mediated through pineal function.

This question is further complicated by the anatomy of the pineal region. The nearby subcommissural organ (SCO) is a specialized secretory structure that releases SCO-spondin (Sspo) into the cerebrospinal fluid, where it assembles into the Reissner fiber (RF). The RF is an extracellular thread that extends through the ventricular system and central canal (Sepúlveda et al., 2021). RF formation requires motile cilia-driven CSF flow (Cantaut-Belarif et al., 2018), while failure to form the RF, either through disruption of motile cilia or Sspo itself, results in spinal curvature in zebrafish (Lu et al., 2020; Rose et al., 2020; Troutwine et al., 2020). Thus, surgical pinealectomy could conceivably disrupt the SCO and RF formation, resulting in spinal curves. Intriguingly, the SCO itself is also subject to serotonergic regulation. Serotonergic fibers innervate SCO ependymal cells in mammals, while experimental manipulation of 5-HT signaling alters SCO secretory activity and Sspo expression in several vertebrate species (Bouchaud et al., 1979; Léger et al., 1983; Sakumoto et al., 1984; Richter et al., 2004). This raises the possibility that serotonergic signaling could influence RF formation or homeostasis. We therefore tested these possibilities by combining targeted chemical ablation of the pineal gland, thereby avoiding the collateral effects of surgery, with direct examination of RF morphology in *tph2* mutants.

To ablate the pineal gland, we generated Tg(*exorh:mCherry-2A-NTR2.0*) zebrafish in which the pineal-specific *exorh* promoter drives both mCherry and the enhanced nitroreductase NTR 2.0 (Asaoka et al., 2002; Sharrock et al., 2022). NTR 2.0 converts the prodrug metronidazole (MTZ) into a cytotoxic metabolite, allowing selective ablation of NTR-expressing cells. Reporter expression was restricted to the pineal gland (Fig. 3A). Transgenic animals were exposed to MTZ continuously from the 1-cell stage to 4 days pf and subsequently retreated for 24 h every 3 days to prevent pinealocyte regeneration. Loss of pineal fluorescence was confirmed throughout the experiment, and complete ablation was verified by immunostaining at early, intermediate, and final time points (Fig. 3A).

We then followed pineal-ablated animals through the period during which spinal curvature emerges in *tph2* mutants. At 1.5 months pf, none of the MTZ-treated NTR+ animals exhibited spinal curvature (0/47; Fig. 3B). In an independent cohort followed to 3.5 months pf, spinal curvature was again absent from all pineal-ablated animals (0/25). Together, these results show that sustained loss of the pineal gland is not sufficient to induce scoliosis in zebrafish.

Because disruption of the RF is sufficient to cause spinal curvature in zebrafish (Lu et al., 2020; Rose et al., 2020; Troutwine et al., 2020), we next asked whether loss of Tph2 might affect spine morphology through this pathway. We visualized the RF in *tph2* mutants using fluorescently tagged SCO-spondin (Sspo-GFP; Troutwine et al., 2020) and followed its morphology through larval development. The RF formed normally and remained continuous in *tph2* mutants through 21 days pf, around the developmental stage at which spinal curves emerge in mutants (Fig. 3C). We detected no differences in RF morphology between mutants and control siblings. This suggests RF defects are not the cause of spinal curves in *tph2* mutants.

Together, these findings argue against the hypothesis that the pineal gland is critical for maintaining spinal morphology. They further show that *tph2*-dependent curvature develops despite an intact RF, excluding two prominent mechanisms as explanations for *tph2*-dependent curvature. Instead, our findings point toward a requirement for serotonergic signaling in other neuronal populations.

### Serotonergic circuitry and locomotion are altered before scoliosis onset

5-HT is an important neuromodulator of vertebrate spinal locomotor circuits which influences the pattern and output of locomotor activity in zebrafish (Schmidt and Jordan, 2000; Brustein et al., 2003; Montgomery et al., 2018). In larval zebrafish, a population of serotonergic intraspinal neurons (ISNs) arise from the ventral floor plate and develop within the spinal cord (Lillesaar et al., 2007; Montgomery et al., 2016; Chen et al., 2023). These persistent ISNs also express *fev* (formerly *pet1*), an ETS-family transcription factor associated with serotonergic neuronal differentiation (Cheng et al., 2003; Lillesaar et al., 2007; Montgomery et al., 2016). Fev+ ISNs provide a local source of spinal 5-HT, extend processes in close association with spinal motor circuitry and directly modulate spinal locomotor output (McLean and Fetcho, 2004; Montgomery et al., 2018). We therefore asked whether loss of *tph2* altered the Fev+ ISN population and whether *tph2* mutants exhibited concomitant changes in locomotor behavior.

To assess the Fev+ ISN population directly, we generated a Tg(*fev:mCherryCAAX*) transgenic reporter line and then quantified Fev+ neurons at 7 days pf. Counts were performed across two consecutive 350 μm regions of the spinal cord, beginning at the first Fev+ ISN and extending 700 μm caudally to approximately the level of the posterior yolk extension. *tph2* mutants contained significantly fewer Fev+ ISNs within the most rostral region compared with wild-type siblings (Fig. 4A-B; *P* = 3.95 × 10^-5^). Fev+ ISN number was also reduced in the adjacent, more caudal region, although the difference was smaller (Fig. 4B; *P* = 0.032). Across the combined 700 μm region of the rostral spinal canal, *tph2* mutants showed a substantial reduction in Fev+ ISN number (Fig. 4C; *P* = 1.42 × 10^-4^). This decrease could reflect either a reduction in ISN number or altered maintenance of *fev* expression in otherwise surviving neurons. Consistent with the latter possibility, *Tph2*-deficient mice exhibit reduced Fev expression in the rostral raphe despite preservation of Lmx1b+ neurons, suggesting that loss of 5-HT can alter maintenance of serotonergic identity without eliminating the underlying neuronal population (Kim et al., 2014). Overall, we show that loss of *tph2* reduces the number of Fev-expressing ISNs during larval development.

To determine whether loss of *tph2* alters locomotor behavior before the onset of overt spinal curvature, we tracked swimming larvae at 15 days pf across three experiments (WT, n=22; *tph2*, n=23). Individual fish were recorded for 10 min in a free-swimming arena, then quantified for a range of locomotion metrics. *tph2* mutants spent significantly more time swimming than wild-type siblings (Fig. 4D). We asked whether this increase reflected changes in the structure of locomotor bouts, defined as discrete episodes of continuous swimming separated by interbout periods of inactivity. Mutant larvae exhibited longer swim bouts (Fig. 4E) and shorter interbout intervals (Fig. 4F). This indicates that mutants both remained active for longer and resumed swimming sooner after stopping. By contrast, average swimming speed was not significantly different between genotypes (Fig. 4G), suggesting that loss of *tph2* altered the temporal organization of locomotion without affecting speed.

We next asked whether this increased activity was accompanied by changes in turning behavior. Because *tph2* mutants spent more time swimming overall, we normalized turning to the amount of time each animal was actively swimming. On this basis, mutants devoted the same proportion of swimming time to turning as wild-type larvae (Fig. 4H). Next, we assessed whether this apparent similarity masked changes in particular classes of turns. However, J-turns (P = 0.965), R-turns (P = 0.531), and C-bends (P = 0.463) were also unchanged. Thus, the increase in locomotor activity was not accompanied by a detectable shift in the frequency or composition of turning behavior.

Because serotonergic signaling is also associated with anxiety-related behavior (Maximino et al., 2013), we asked whether the increased locomotor activity in *tph2* mutants was accompanied by altered edge-seeking behavior, termed thigmotaxis (Richendrfer et al., 2012). We therefore quantified the proportion of time that larvae spent near the edge of the arena. However, *tph2* mutants did not differ from wild-type siblings in time spent at the edge (Fig. 4I). This suggests that the locomotor phenotype is not related to changes in spatial preference within the arena.

Overall, these findings demonstrate that loss of *tph2* shifts larvae toward a more persistent state of active locomotion, characterized by longer swimming bouts and shorter periods of inactivity, without changing the type of locomotion. This is consistent with previous work linking serotonergic signaling to suppression of locomotor activity in zebrafish (Brustein et al., 2003; Montgomery et al., 2018). Moreover, dorsal raphe serotonergic neurons are preferentially active during spontaneous quiescence, and their activation can promote a quiescent behavioral state (Qi et al., 2026). In agreement, *tph2* mutants have also been shown to exhibit increased waking activity and reduced sleep at 5 days pf (Oikonomou et al., 2019). Together with these results, our findings therefore suggest that, in the absence of 5-HT, larvae are less likely to enter or remain in quiescent states and instead spend more time engaged in locomotor activity.

Importantly, these findings demonstrate an altered locomotor phenotype shortly before the first appearance of overt spinal curvature in *tph2* mutants. During this period, the juvenile spine is still growing rapidly and is likely especially sensitive to the forces generated by repeated axial muscle contraction. Increased locomotion, with longer bouts of activity and shorter periods of rest, would therefore be expected to increase the mechanical demands placed on this immature vertebral column. We therefore propose that loss of *tph2* alters the physical environment in which the spine develops, exposing a growing and still vulnerable axis to greater and more sustained mechanical loading. Over time, these repeated demands may push the system beyond its ability to maintain alignment, increasing susceptibility to progressive curvature. An additional possibility is that altered serotonergic signaling affects the fine control of axial posture. Zebrafish continuously use differential activation of dorsal and ventral axial motor circuits to correct body orientation (Bagnall and McLean, 2014). Thus, loss of *tph2* could alter the pattern of axial muscle activation used to maintain body alignment. These results cohere with recent findings of other pathways that converge on motor output and contribute to spinal curvature. Disruption of glycinergic neurotransmission in *slc6a9* mutant zebrafish causes abnormal left-right neural activity and asymmetric axial muscle contraction (Wang et al., 2024). Furthermore, disruption of EphrinB3-Epha4 signaling impairs spinal central pattern generator organization and produces abnormal locomotor coordination before the development of scoliosis (Wang et al., 2025). Together with our findings, these studies collectively support a model in which relatively subtle changes in the neural control of axial musculature can alter the mechanical forces repeatedly experienced by the growing vertebral column and thereby increase susceptibility to progressive curvature.

Overall, our findings identify neuronal serotonergic signaling as an important regulator of spinal morphology during growth. Loss of *tph2* produces progressive spinal curvature after initially normal vertebral development. We demonstrate that pineal- and Reissner fiber-associated pathways are unlikely to account for the curvature phenotype. Instead, *tph2* mutants show changes in spinal serotonergic circuitry together with altered locomotor behavior before overt curvature develops. We therefore favor a model in which serotonergic dysfunction changes the motor and mechanical environment experienced by the growing vertebral column, through increased locomotor activity, altered postural control, or a combination of both.

This may help explain longstanding associations between serotonergic pathways and human AIS. It is also consistent with a broader literature implicating altered neuromuscular and postural control in AIS (Dufvenberg et al., 2018; Lau et al., 2022; Ng et al., 2022; Chan et al., 2024). Such a mechanism could be particularly important during periods of rapid adolescent growth, when relatively small biases in muscle activity or loading may accumulate over time into three-dimensional curves. Determining which serotonergic populations and downstream motor circuits are required for spinal stability, and whether comparable alterations in neuromuscular control precede curve onset in humans, will be important next steps toward understanding how neural activity and skeletal growth become coupled during scoliosis development.

## EXPERIMENTAL PROCEDURES

### Zebrafish husbandry.

AB and TL strains of *Danio rerio* were used. Adult zebrafish were maintained under standard laboratory conditions in a recirculating aquatic system at 28.5°C on a 14-hr light/10-hr dark cycle. Animals were maintained in an AAALAC International accredited facility. All procedures were approved by the University of Oregon Institutional Animal Care and Use Committee under protocol 21–45.

### Zebrafish lines.

The transgenic and mutant zebrafish lines used in this study were Tg(*fev:mCherry-CAAX*)^*b1533*^, Tg(*exorh:mCherry-P2A-NTR2.0*)^*b1516*^, *tph2*^*ct817*^ (Oikonomou et al., 2019), TgBAC(*entpd5a:killerRed*)^*hu7478Tg*^ (Wopat et al., 2018) and *sspo-gfp*^*ut24*^ (Troutwine et al., 2020). Experiments were performed on zebrafish ranging from embryonic stages to 1 year of age. Sex was not determined for experiments performed at embryonic or larval stages. For juvenile and adult experiments in which sex could be determined, both male and female animals were included, and sex-specific results are reported where applicable.

### Cloning and transgenesis.

The construct used to generate the Tg(*exorh:mCherry-P2A-NTR2.0*)^*b1516*^ transgenic line was assembled using Gateway recombination cloning (Hartley et al., 2000; Cheo et al., 2004; Kwan et al., 2007). The mCherry-P2A-NfsB_VvF70A/F108Y coding sequence, hereafter referred to as mCherry-P2A-NTR2.0, was amplified from Addgene plasmid #158653 (Sharrock et al., 2022) using Phusion High-Fidelity DNA Polymerase (New England Biolabs) and the following primers: forward, 5′-GGGGACAAGTTTGTACAAAAAAGCAGGCTTTgccaccatggtgagcaaggg-3′; and reverse, 5′-CCACTTTGTACAAGAAAGCTGTCCCCCTAGTTttagatttcggtaaaaacag-3′ (Integrated DNA Technologies). Uppercase nucleotides indicate *attB*-containing adaptor sequences, and lowercase nucleotides indicate template-specific sequences. The PCR product was purified and recombined into pDONR221 using Gateway BP Clonase II Enzyme Mix (Thermo Fisher Scientific) to generate the middle entry clone pME-mCherry-P2A-NTR2.0. Multisite Gateway cloning was then performed using the pCK011 p5E-exorh promoter entry clone (Addgene plasmid #195949; Kemmler et al., 2023), pME-mCherry-P2A-NTR2.0, and p3E (Kwan et al., 2007). These entry clones were recombined into the Tol2 destination vector pDestTol2pACryGFP (Addgene plasmid #64022; Berger et al., 2013) using Gateway LR Clonase II Enzyme Mix (Thermo Fisher Scientific). The resulting exorh Tol2 construct was used for transgenesis.

The construct used to generate the Tg(*fev:mCherry-CAAX*)^*b1533*^ transgenic line was also assembled using Gateway recombination cloning. Multisite Gateway cloning was performed using the p5E-MCS-Fev promoter entry clone, pME-mCherryCAAX, and p3E (Kwan et al., 2007). These entry clones were recombined into the Tol2 destination vector pDESTTol2pA2 using Gateway LR Clonase II Enzyme Mix (Thermo Fisher Scientific). Complete plasmid sequences were verified by whole-plasmid sequencing (Azenta Life Sciences or Plasmidsaurus).

### Zebrafish line generation.

Zebrafish lines for Tg(*fev:mCherry-CAAX*) and Tg(*exorh:mCherry-P2A-NTR2.0*) were generated by injecting 100 ng/μL of the corresponding plasmid together with 25 ng/μL *tol2* transposase mRNA. Transgenic larvae were identified at 3 days pf by fluorescence in the relevant tissue and, for the *exorh* line, by the secondary eye marker. Fluorescent larvae were raised to 3 months pf and outcrossed to AB fish. F1 progeny were again screened for fluorescence, raised to 3 months pf, and outcrossed to AB fish. Before use in experiments, F2 clutches were confirmed to contain approximately 50% fluorescent embryos, consistent with transmission of a single transgene insertion. Lines were subsequently maintained by outcrossing to AB fish, with transgenic animals identified by fluorescence at 3 days pf or by genotyping from fin clips at 1 month pf or older.

### Quantitation of standard length.

Fish were imaged during larval stages using a Leica S9i stereomicroscope with an integrated 10-megapixel camera. During juvenile and adult stages, fish were imaged using a mounted Google Pixel 9 Pro 50-megapixel camera. A ruler was included in each image for spatial calibration, and standard length was measured in ImageJ from the tip of the snout to the caudal peduncle. Because individual fish were not tracked longitudinally across time points, standard length was analyzed using an ordinary two-way ANOVA.

### 5-HT treatment.

Wild-type AB zebrafish embryos were exposed to exogenous 5-HT by adding 1 or 2 mM 5-HT to the rearing water from the 1-cell stage until 6 days pf. Mock controls from the same clutch were raised in parallel. 5-HT-containing water was replaced daily. At the end of the treatment, 5-HT was removed by three washes with fish water. Animals were then raised to 2.5 months pf for skeletal analysis.

### Chemical ablation.

After establishment of a stable Tg(*exorh:mCherry-P2A-NTR2.0*) transgenic line, transgenic fish were outcrossed to wild-type AB fish. One hundred 1-cell-stage embryos from the resulting clutch were transferred to embryo medium containing 1 mM metronidazole (MTZ). The working solution was prepared by diluting a 50 mM MTZ stock containing 1% DMSO 1:50 in embryo medium. At 18 hr pf, embryos were dechorionated and transferred to fresh embryo medium containing 1 mM MTZ. Embryos were treated continuously with MTZ until 4 days pf and subsequently retreated for 24 hr every 3 days. Pineal ablation was confirmed throughout the experiment by assessing mCherry fluorescence directly and through immunostaining for mCherry and 5-HT.

### Faxitron imaging.

X-ray images were acquired using a Faxitron UltraFocus/MultiFocus X-ray imaging system (Faxitron Bioptics). Fish were imaged either immediately after euthanasia or following fixation in 4% paraformaldehyde for up to two weeks. Depending on animal size, fish were positioned within the imaging chamber and imaged using the instrument’s microfocus X-ray source. The voltage was set to 25 kV. Exposure time and magnification were adjusted as needed to obtain sufficient contrast and resolution for visualization of the vertebral column.

### X-ray micro-computed tomography (μCT).

Whole body μCT scans were acquired using a vivaCT 80 system (Scanco Medical) at 18.5 μm voxel resolution for fish aged >1.5 months pf, or at 10 μm voxel resolution for 1 month pf fish, as previously described (Bearce et al., 2022). High resolution scans of individual vertebrae were acquired using a Zeiss Xradia 620 Versa μCT system at 3 μm voxel resolution. Digital dissections of the spinal column were performed in 3D Slicer using the Segment Editor, and subsequent analyses were conducted as previously described (Bearce et al., 2022; Bearce et al., 2023).

### Quantitation and statistical analysis of spinal curvature.

Vertebral coordinates were aligned using the approach described by Voigt et al. (2026), and spinal shape was compared between genotypes in the dorsoventral (DV) and mediolateral (ML) planes. Each fish was treated as a single biological replicate. Analyses included vertebral positions 2–24 as positions 1 and 25 were excluded because they are constrained by the alignment procedure. DV displacement was analyzed using the aligned DV coordinate (final_y), while ML curvature was analyzed using absolute ML displacement (|final_z|), as there was no evidence of a left-right bias in curve direction. The dataset contained 10 wild type and 10 *tph2* mutant fish, with five males and five females of each genotype.

For whole spine comparisons, displacement at each vertebral position was modeled as a function of genotype and sex. The genotype effects across all 23 vertebral positions were squared and summed to generate a single measure of overall spinal shape difference. Significance was assessed by exact permutation of genotype labels within sex, giving 63,504 possible genotype assignments. The whole-spine statistic was recalculated for each assignment to generate the null distribution, and the permutation P value was calculated as the proportion producing a statistic at least as large as the observed value. Sex effects were assessed similarly by permuting sex within genotype. Genotype-by-sex interactions were tested by residual permutation after fitting an additive model containing both genotype and sex.

To identify individual regions of the spine that differed between genotypes, we used a max-T permutation test. At each vertebral position, displacement was modeled as a function of genotype and sex and a t statistic was calculated for the genotype effect. Genotype labels were then permuted within sex across whole fish, and the largest absolute t statistic observed across the 23 positions was retained for each permutation. These values were used to calculate multiple comparison-adjusted P values for each vertebral position.

Difference plots show the estimated genotype effect at each vertebral position, expressed as mutant minus wild type displacement. Simultaneous 95% confidence intervals were calculated from the max-T permutation distribution. Vertebral positions were considered significantly different between genotypes when the confidence interval excluded zero.

### Immunofluorescence.

Embryos were fixed overnight at 4°C in 4% paraformaldehyde with gentle rocking. Following fixation, embryos were washed in phosphate buffered saline (PBS), serially dehydrated to 100% methanol, and stored at -20°C. Before staining, embryos were rehydrated and blocked for 2 hr in blocking solution containing 5% normal sheep serum (NSS), 1% DMSO, and 0.1% Tween-20 in 1× PBS. Primary antibodies were diluted 1:500 in PBS and incubated with embryos overnight at 4°C with rocking. Embryos were then washed five times for 30 min in rinse solution containing 1% NSS, 1% DMSO, and 0.1% Tween-20 in 1× PBS. Secondary antibodies were diluted 1:1000 in rinse solution and incubated with embryos overnight at 4°C with rocking, protected from light. Embryos were then washed five times for 30 min in rinse solution, protected from light, followed by a final 30-min wash in 0.1% Tween-20 in 1× PBS before mounting for imaging.

### Confocal microscopy.

Live imaging of Sspo-GFP was performed as previously described (Bearce et al., 2022). Immunofluorescence imaging was performed using a Zeiss LSM 880 confocal microscope with 10×, 20×, and 40× water-dipping objectives.

### Locomotor behavior.

Locomotor behavior was assessed at 15 days pf using a behavioral recording and analysis pipeline adapted from (Desban et al., 2026). Individual zebrafish were placed in shallow circular arenas (5 cm diameter, 2 mm depth) containing system water and allowed to habituate for 5 min. Fish were then recorded from above for 10 min at 25 frames per second under homogeneous illumination using an infrared imaging system.

Fish body position was tracked using ZebraZoom (Mirat et al., 2013), which identifies a series of points along the body in each video frame. Tracking data were subsequently analyzed using custom Python scripts as described in (Desban et al., 2026). Fish were classified as swimming when instantaneous speed exceeded 9 mm/s, and locomotor activity was quantified as the fraction of recording time spent swimming. Individual swimming bouts were identified from transitions between moving and stationary periods, from which mean bout duration and interbout interval were calculated. Mean swimming speed was calculated from periods in which fish were actively swimming. Turning behavior was quantified from changes in body heading between successive frames. Full details are provided in (Desban et al., 2026).

## Figures and Tables

**Fig. 1 |:**
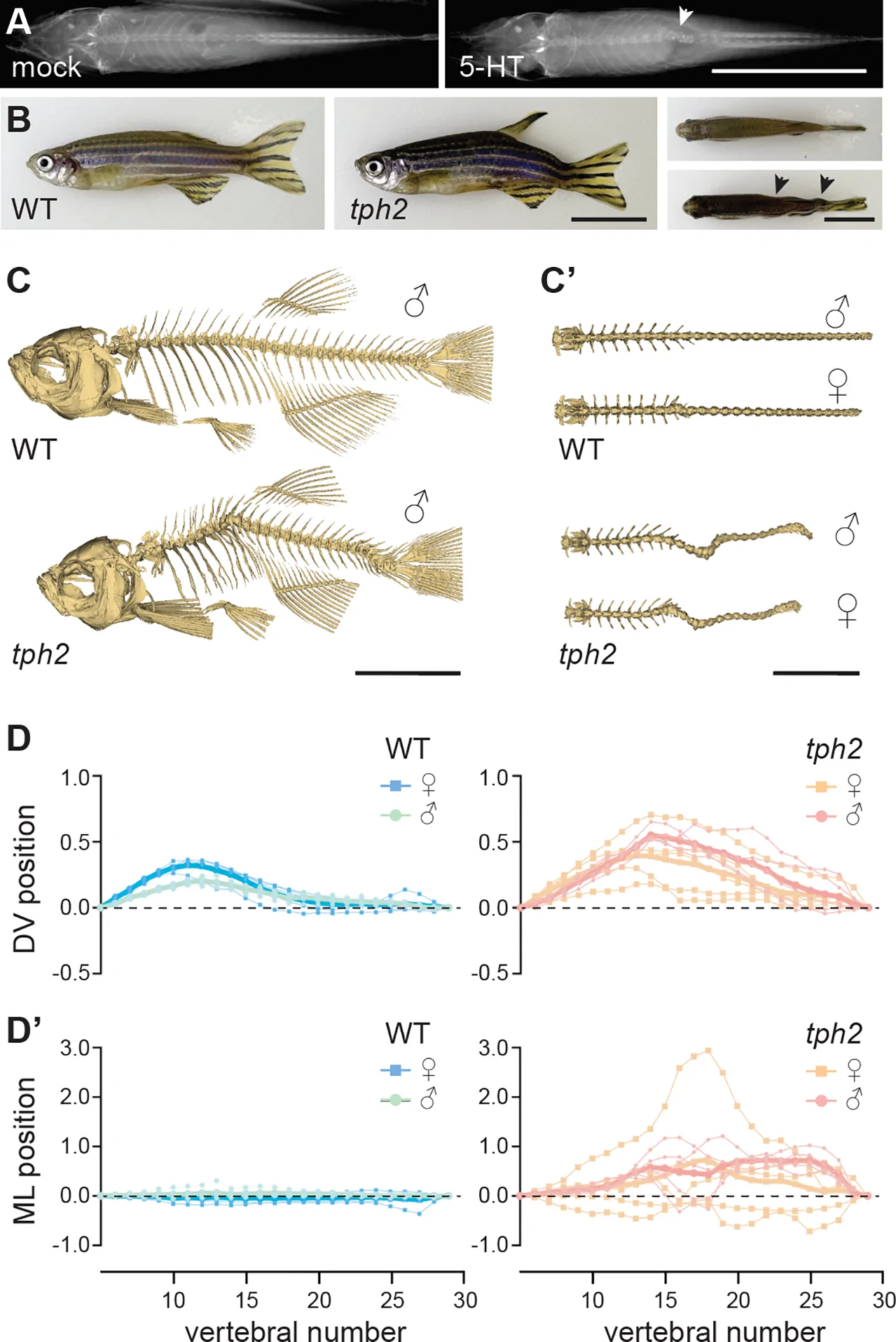
*tph2* mutants exhibit abnormal spine morphology. **(A)** 2.5 months pf wild-type fish either mock treated or treated with 2 mM 5-HT from 0–6 days pf. Treated fish developed spinal curvature (arrowhead). **(B)** Adult wild-type and *tph2* mutant males (6 months pf) shown in lateral and dorsal views. Arrows mark the positions of lateral spinal curvatures in *tph2* mutants. **(C-C’)** Lateral (C) and dorsal (C’) views of three-dimensional reconstructions from μCT scans of wild-type and *tph2* mutants at 3 months pf. **(D-D’)** Quantification of spinal shape from aligned vertebral coordinates in wild type and *tph2* mutants at 3 months pf. Dorsoventral (DV) and mediolateral (ML) vertebral positions are plotted along the length of the spine. Positions are shown in arbitrary units and represent relative displacement of each vertebra from the reference spinal axis. Each line represents an individual fish; females and males are indicated separately. Thicker lines show means for each sex. n = 10 fish per genotype. Scale bars: 10 mm (A-C).

**Fig. 2 |:**
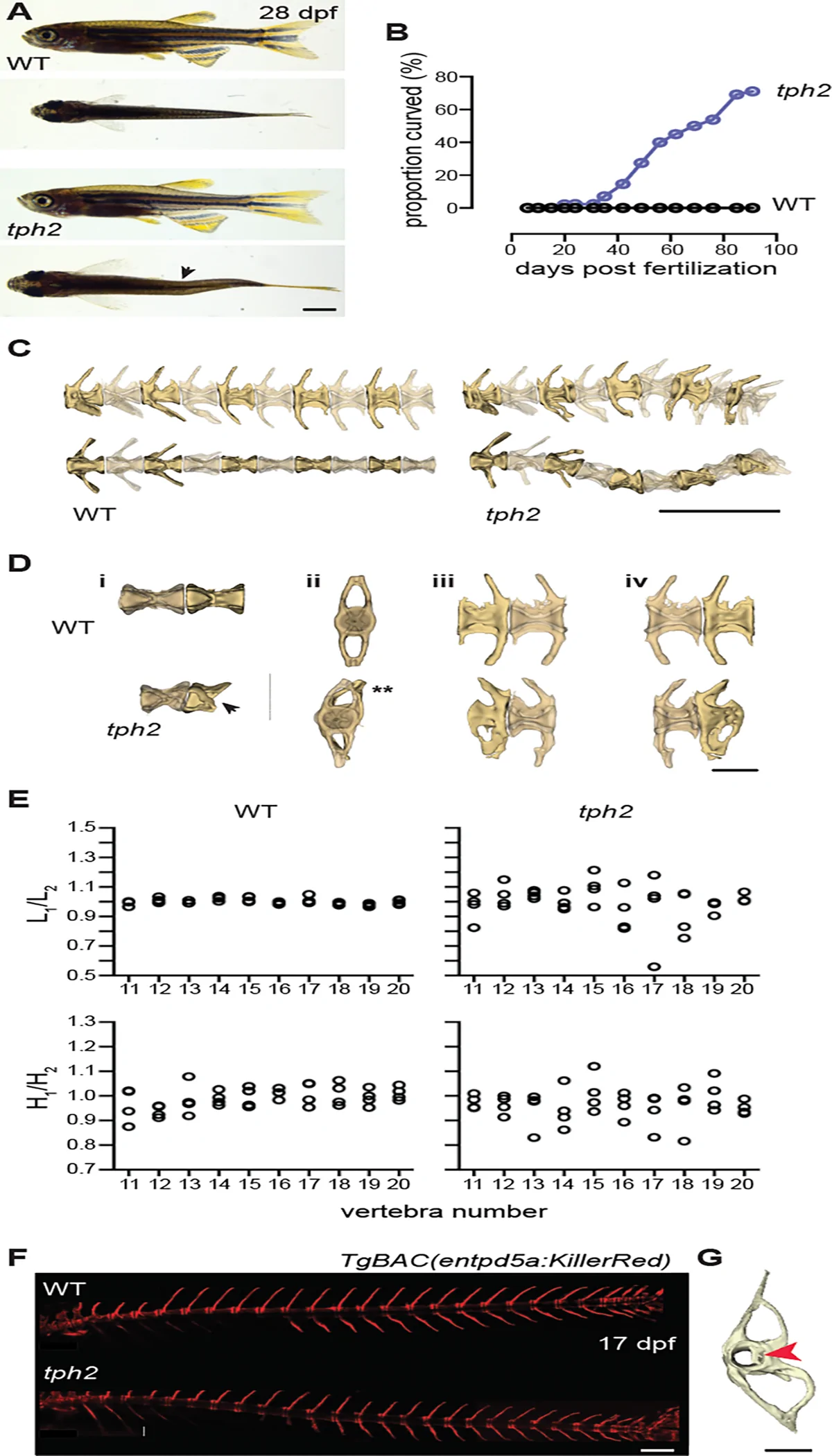
Spinal curvature in *tph2* mutants arises during juvenile growth after normal vertebral patterning. (A) Representative lateral and dorsal brightfield images of WT and *tph2* mutant zebrafish at 28 days pf. The arrowhead marks a spinal curve in the *tph2* mutant. (B) Developmental onset of spinal curvature. Fish were examined during juvenile growth and scored for the presence of visible spinal curvature. Curves first became apparent at approximately 20 dpf in some fish then increased in penetrance during subsequent growth. (C) Representative three-dimensional reconstructions of isolated 6 months pf WT and *tph2* mutant spines generated from μCT datasets, shown in lateral (top) and dorsal (bottom) views. *tph2* mutant spines show three-dimensional curvature and localized vertebral distortion. (D) Representative vertebrae from 6 months pf WT and *tph2* mutant animals illustrating abnormalities associated with curved regions. Compared with WT vertebrae, mutant vertebrae show wedge shapes (i), twisting (ii), compression (iii) and occasional fusion between adjacent vertebrae (iv). (E) Quantification of vertebral morphology across vertebrae 11–20 in WT and *tph2* mutants. Ratios of longitudinal dimensions (L1/L2; top) and dorsoventral dimensions (H1/H2; bottom) are plotted for individual vertebrae. Vertebrae from *tph2* mutants show increased morphological variability, consistent with local vertebral distortion. (F) Representative images of TgBAC(*entpd5a:KillerRed)* in WT and *tph2* mutants at 17 days pf (standard length: 8.36 mm for WT and 8.49 mm for *tph2*). Mineralizing domains of the notochord sheath show regular segmentation in both *tph2* mutants and controls before overt spinal curvature. (G) Representative high-resolution μCT reconstruction of a vertebra from a *tph2* mutant examined shortly after curve onset. Arrowhead marks small ectopic mineralized fragment. Scale bars: 1 mm (A), 250 μm (D, F-G).

**Fig. 3 |:**
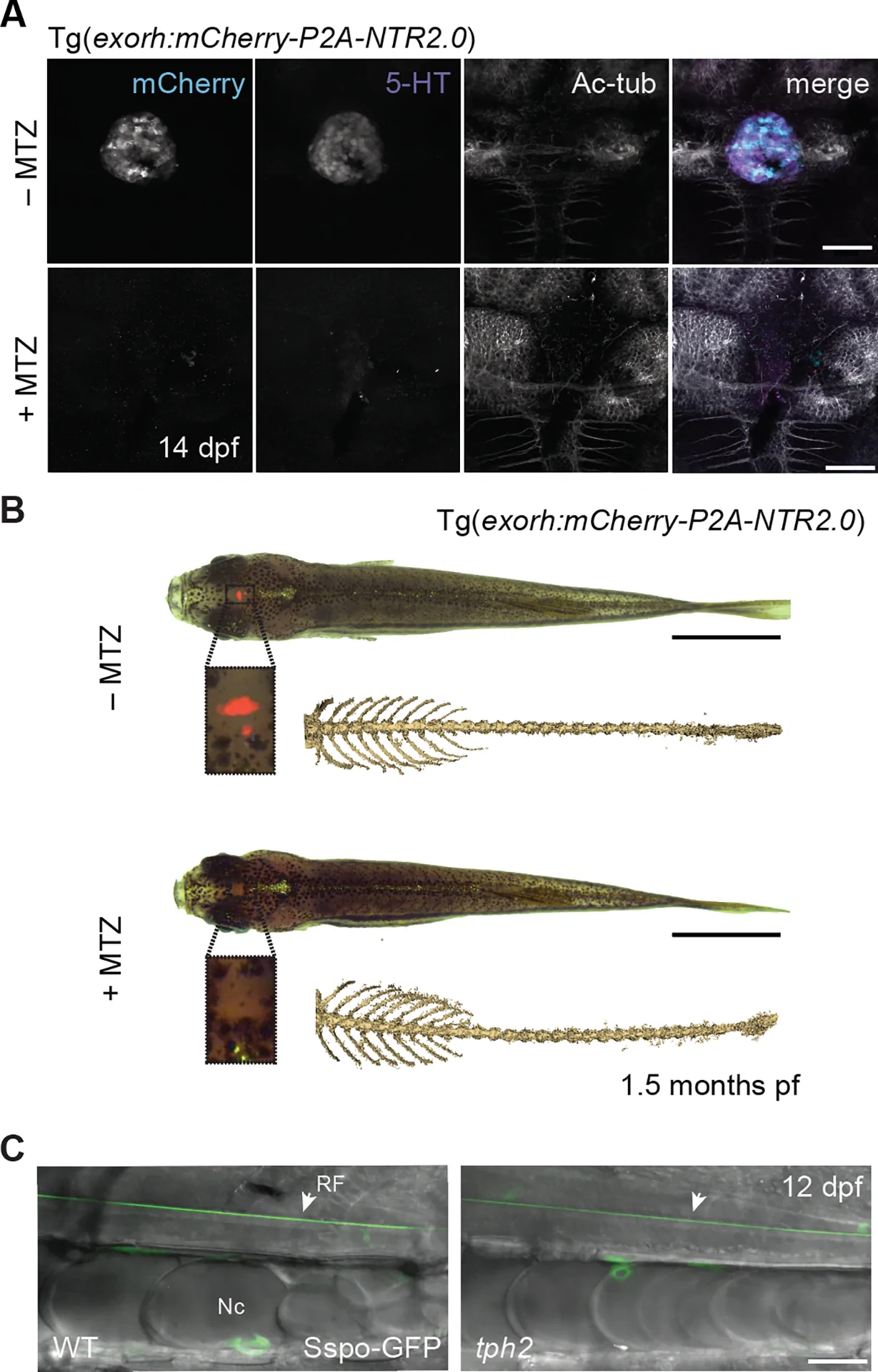
Pineal ablation does not induce spinal curvature and the Reissner fiber remains intact in *tph2* mutants. (A) Representative images of Tg(*exorh:mCherry-P2A-NTR2.0*) zebrafish at 14 days pf following repeated treatment with metronidazole (MTZ). Pinealocytes were visualized by mCherry fluorescence and 5-HT immunostaining. Acetylated α-tubulin (Ac-tub) marks surrounding neural structures. Untreated transgenic animals show robust mCherry and 5-HT signal in the pineal gland, whereas MTZ-treated animals show loss of both signals, confirming sustained pineal ablation. (B) Representative dorsal views of Tg(*exorh:mCherry-P2A-NTR2.0*) animals at 1.5 months pf with or without MTZ treatment. Insets show the pineal region. μCT reconstructions are shown alongside each fish. Despite sustained loss of the pineal gland, MTZ-treated fish developed normally aligned vertebral columns without spinal curvature. (C) Representative images of the RF in 12 days pf WT and *tph2* mutant larvae carrying *sspo-gfp*. Sspo-GFP labels the RF (arrowheads), which remains continuous in *tph2* mutants. Nc, notochord. Scale bars: 50 μm (A), 0.5 mm (B), 40 μm (C).

**Fig. 4 |:**
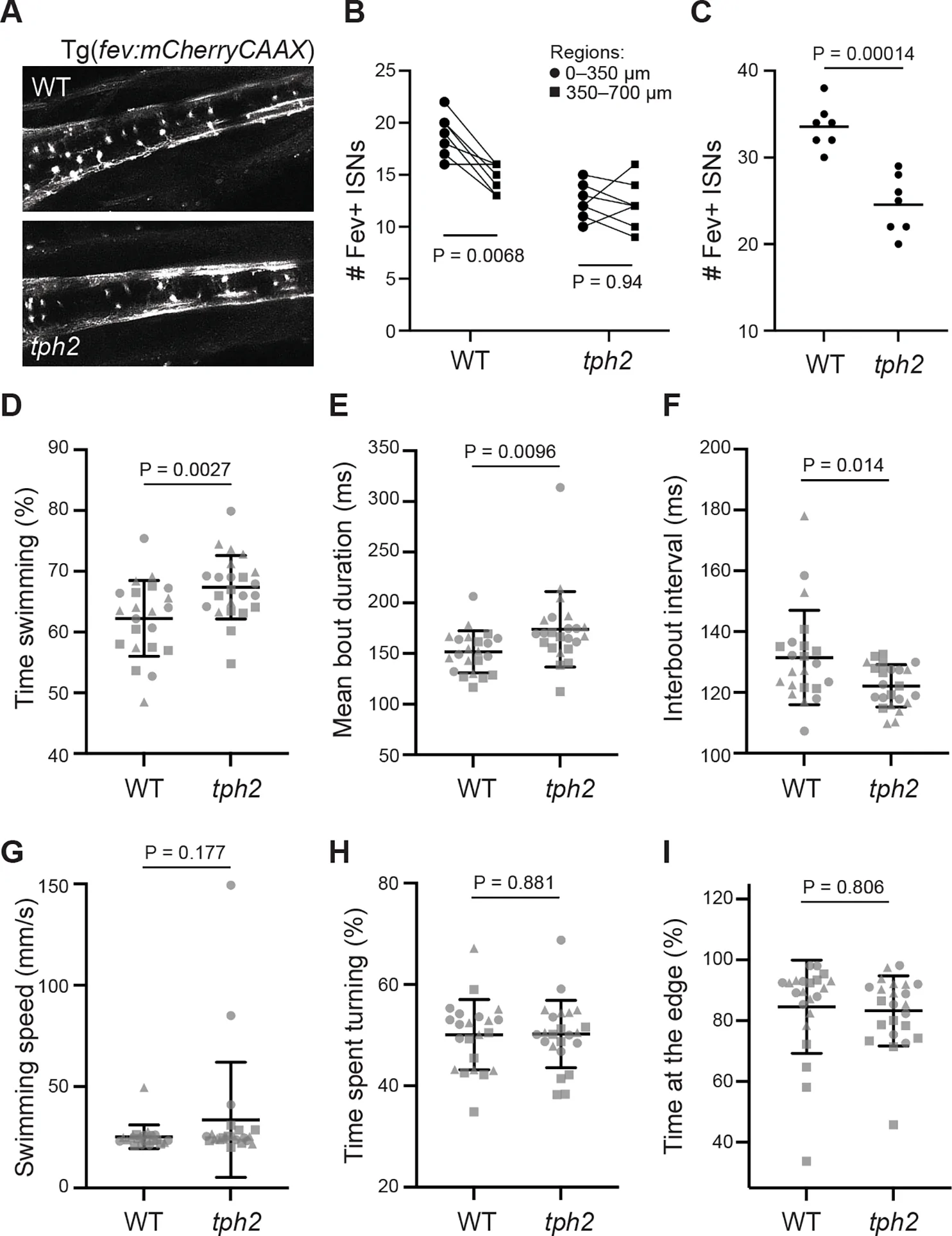
Serotonergic intraspinal neurons and locomotor behavior are altered in *tph2* mutants before spinal curvature. (A) Representative images of Tg(*fev:mCherryCAAX*) expression in the rostral spinal cord of 7 days pf WT and *tph2* mutant larvae. (B) Fev+ ISNs were quantified in two consecutive 350 μm regions extending caudally from the first Fev+ ISN. Connected points represent counts from the two regions within the same animal. Two-way repeated measures ANOVA revealed significant effects of genotype (*P* = 0.00011) and region (*P* = 0.0042). Šídák corrected comparisons showed significantly more ISNs in the 0–350 μm than the 350–700 μm region in WT (*P* = 0.0068) but not in *tph2* mutants (*P* = 0.94). (C) Total number of Fev+ ISNs across the combined 700 μm region was reduced in *tph2* mutants (*P* = 0.00014, Welch’s *t*-test). (D-I) Locomotor behavior was quantified at 15 days pf before the onset of overt spinal curvature. Individual animals were recorded during free swimming and analyzed across three independent experiments, denoted by distinct symbols. Horizontal bars show mean ± SD. Statistical comparisons were performed using two-way ANOVA.

**Resources:** 

REAGENT or RESOURCE	SOURCE	IDENTIFIER
Antibodies		
Anti-Serotonin antibody produced in rabbit	Sigma	S5545
Monoclonal Anti-Tubulin, Acetylated antibody produced in mouse	Sigma	T6793
Anti-mCherry antibody [1C51]	Abcam	ab125096
Anti-GFP antibody	Abcam	ab13970
Goat anti-Chicken IgY (H+L) Secondary Antibody, Alexa Fluor^™^ 488	ThermoFisher	A-11039
Goat anti-Rabbit IgG (H+L) Highly Cross-Adsorbed Secondary Antibody, Alexa Fluor^™^ 546	ThermoFisher	A-11035
Goat anti-Mouse IgG (H+L) Cross-Adsorbed Secondary Antibody, Alexa Fluor^™^ 647 - A-21235	ThermoFisher	A-21235
Bacterial and virus strains		
NEB 10 beta Competent *E. coli*	New England Biolabs	C3019H
Experimental models: Organisms/strains		
*Danio rerio* (AB and TU)	Zfin	AB/TU
SSPO:GFP knock in		
Software and algorithms		
SLEAP	Talmo Lab, sleap.ai	
R	R-project.org	
ImageJ	imagej.net	
3D Slicer	Slicer.org	
